# INCLUSION OF FAMILY MEMBERS INTO CONVERSATIONS ABOUT AGING RESEARCH: IMPORTANCE OF INTERGENERATIONAL INFLUENCE

**DOI:** 10.1093/geroni/igad104.2921

**Published:** 2023-12-21

**Authors:** Trudy Gaillard, Donna Neff, Cynthia Morton, Phildra Swagger, Fern Webb

**Affiliations:** Florida International University, Miami, Florida, United States; University of Central Florida, Orlando, Florida, United States; University of Florida, Gainesville, Florida, United States; University of Central Florida, Orlando, Florida, United States; University of Florida, Gainesville, Florida, United States

## Abstract

Identifying effective strategies to increase recruitment and retention of culturally diverse adults (African Americans [AA], Caribbean [CN] and Hispanic/Latino [HL]) into aging research is a public health priority. Intergenerational influence (IGI), defined as the “influence of one generation on another in terms of the transfer of skills, attitudes, preferences, values, and behaviors” (Shah & Mittal, 1997), was used to engage AA, CN and HL adults ages 25+ into conversations about aging research. We recruited AA, CN, and HL adults ≥ 65 years and a family member/friend between 25-64 years to participate in virtual listening sessions (LS). A semi-structured guide was used to discuss IGI, attitudes, beliefs, and perspectives of research. All LS were recorded and transcribed verbatim. NVivo software was used for data management and analysis. The constant comparative method was used for analysis. A total of 134 LS was conducted with African American [N=131], Caribbean [N= 112] and Hispanic/Latino [118] adults, representing 93 males and 268 females. We found three major themes highlighting the importance of IGI: 1) transfer of cultural knowledge; 2) benefit of future generations and 3) the lived experiences. The importance of IGI and the inclusion of family members and friends into discussions about healthy aging and research participation was a consistent theme among all culturally diverse groups. Findings suggest that discussing research participation with family and friends can enhance research participation of older adults. Researchers should develop IGI strategies that include family and friends (i.e., younger trusted sources) into recruitment design of aging research.

